# Aquaticity as a Latent Dimension of Aquatic Performance: Conceptual Framework and Application to Breath-Hold Diving

**DOI:** 10.3390/jfmk11010120

**Published:** 2026-03-16

**Authors:** Ivan Drviš, Dario Vrdoljak, Nikola Foretić, Željko Dujić

**Affiliations:** 1 Faculty of Kinesiology, University of Zagreb, 10000 Zagreb, Croatia; ivan.drvis@kif.unizg.hr; 2 Centre for Heart, Lung and Vascular Health, University of British Columbia, Okanagan Campus, Kelowna, BC V1V 1V7, Canada; dariov.vrdoljak@ubc.ca; 3 Faculty of Kinesiology, University of Split, 21000 Split, Croatia; nikola.foretic@kifst.eu; 4 Department of Integrative Physiology, School of Medicine, University of Split, 21000 Split, Croatia

**Keywords:** aquaticity latent construct, aquatic sports performance, exercise physiology, sport biomechanics, neuromuscular control

## Abstract

Sports performance in aquatic environments is governed by biomechanical, physiological, neuromuscular and perceptual–mental constraints that differ fundamentally from those encountered on land. As a result, athletes with comparable general physiological or motor capacities may achieve markedly different performance outcomes in aquatic sports. Within functional kinesiology and sport science, aquatic performance is still frequently interpreted through isolated physiological, biomechanical, or technical variables, which limits both explanatory depth and applied relevance. This Perspective article introduces aquaticity as an integrated latent construct representing a multidimensional determinant of sports performance specific to the aquatic environment. Aquaticity is conceptualized as a functional framework that modulates how general physiological and motor capacities are expressed under aquatic constraints, integrating key domains of exercise physiology, sport biomechanics, neuromuscular control, energetic regulation, and perceptual–mental stability. The relative contribution of these domains is considered discipline-specific and dependent on task and environmental demands. Breath-hold diving is presented as a particularly suitable model for examining aquaticity, as apnea and hypoxic–hypercapnic stress amplify interactions between physiological regulation, neuromuscular control, and biomechanical efficiency. Training and diagnostic tasks performed in real aquatic settings are interpreted as manifest indicators of aquaticity, enabling ecologically valid athlete monitoring and performance assessment. Within this framework, energetic aquaticity is highlighted as a central functional sub-construct linking metabolic regulation, movement efficiency, and neural control during performance under respiratory constraints. The proposed conceptual framework has important implications for functional kinesiology, sport biomechanics, exercise physiology, and applied athlete monitoring in aquatic sports. Aquaticity is advanced not merely as a descriptive concept, but as a unifying framework that can guide future experimental research, discipline-specific diagnostics, individualized training design, and safety-oriented performance assessment in aquatic environments.

## 1. Introduction

Sports performance in an aquatic environment represents a specific form of human motor activity in which general physical, functional, and conditioning capacities of athletes are expressed under biomechanical, physiological, and perceptual–mental conditions that differ substantially from those encountered on land. Movement in water is characterized by increased resistance, altered force interactions, buoyancy-mediated reduction in net hydrostatic loading, modified sensorimotor feedback, and, in certain disciplines, by breathing constraints and hypoxic–hypercapnic stress. Consequently, athletic performance in water cannot be adequately explained solely through classical land-based models of motor and physiological capacities.

Despite the long tradition of aquatic sports such as swimming, breath-hold diving, finswimming, and spearfishing, scientific research in these domains has most often focused on isolated performance determinants [1,2]. Particular emphasis has been placed on aerobic and anaerobic capacity, movement technique, respiratory physiology, and selected reflexive and autonomic responses of the organism. Although such approaches have yielded valuable insights, their predictive value for actual sports performance in aquatic environments has frequently proven limited, particularly when sport-specific hydrodynamic efficiency and technical execution are not taken into account [3,4]. Numerous studies, as well as practical experience from the training process, indicate that athletes with comparable or even superior physiological potential measured on land may achieve markedly different results in water [5].

In coaching and professional practice, this discrepancy between general capacities and performance in water is often described using informal and partial terms such as “feel for the water,” “technical ease,” or, in the context of breath-hold diving, “large lungs.” Although intuitively meaningful, such expressions capture only isolated aspects of a highly complex adaptive process. As such, they are insufficient for scientific analysis, standardized diagnostics, or systematic planning and evaluation of training processes in aquatic sports. Consequently, kinesiology and sport science still lack a unified conceptual framework capable of integrating the full spectrum of human adaptation to movement and immersion in the aquatic environment.

The term *aquaticity* occasionally appears in professional and scientific communication precisely as an attempt to describe this specific adaptation to the water environment. However, its use to date has been largely descriptive, insufficiently grounded in theory, and weakly linked to concrete performance outcomes in sport. Although individual attempts have been made to define and measure aquaticity scientifically, these approaches have not fully integrated biomechanical, physiological, neurophysiological, and perceptual–mental aspects of performance, nor have they adequately addressed discipline-specific differences among various aquatic sports [6,7].

Due to these limitations, there is a clear need for a more rigorous conceptualization of aquaticity as a scientifically relevant variable. Rather than viewing aquaticity as a collection of isolated skills or abilities, the present work is based on the assumption that aquaticity represents a latent, multidimensional dimension of sports performance specific to the aquatic environment. This dimension modulates how athletes utilize their general physiological and motor capacities in water and helps explain discrepancies between potential assessed by standard tests on land and actual performance under aquatic conditions.

Therefore, the aim of this paper is to conceptually define aquaticity as an integrated latent dimension of sports performance in aquatic environments, grounded in physiological, motor, functional–metabolic, biomechanical, and perceptual–mental adaptations.

Within the framework of functional kinesiology, aquaticity can be understood as an integrative construct that connects sport biomechanics, exercise physiology, neuromuscular control, and applied athlete monitoring, emphasizing functional performance under the specific constraints of the aquatic environment.

In addition, the paper aims to demonstrate the practical and empirical applicability of this concept using breath-hold diving as an example, a sport in which environmental constraints, hypoxic stress, and safety considerations are particularly pronounced. In doing so, this work seeks to establish a bridge between theoretical framework development, scientific diagnostics, and real-world training practice in aquatic sports.

### 1.1. Conceptual and Etymological Basis of Aquaticity

The term *aquaticity* derives from the Latin *aqua* (water) and denotes the degree to which a terrestrial organism is functionally adapted to operate within an aquatic medium. In contrast to morphological adaptations observed in fully aquatic species, such as hydrodynamic body shape or permanent respiratory modifications, human interaction with water is characterized by a predominantly functional and context-dependent form of adaptation. As a terrestrial species, the human motor and physiological systems have evolved under conditions of gravity-dominated locomotion and continuous access to atmospheric oxygen, resulting in sensorimotor and energetic control strategies optimized for movement on land.

Immersion in water introduces a qualitatively different set of environmental constraints, including buoyancy-mediated redistribution of mechanical load, increased fluid resistance, altered proprioceptive and vestibular input, and, in certain aquatic activities, voluntary or task-imposed restriction of breathing. These constraints fundamentally modify the relationships between force production, movement coordination, energetic cost, and physiological regulation, thereby requiring a context-specific reorganization of motor control and metabolic resource deployment.

Within this framework, aquaticity is conceptualized not as an inherent skill or isolated ability, but as a latent dimension reflecting the degree of functional adaptation of the organism–environment system to aquatic constraints. This interpretation parallels the use of constructs such as altitudinality in terrestrial sport science, where exposure to hypobaric or hypoxic environments induces task-relevant physiological and regulatory adaptations that modulate performance independently of baseline motor capacity. Accordingly, aquaticity represents the extent to which general physiological and motor capacities can be effectively expressed under immersion-related constraints, without implying permanent morphological transformation of the human organism.

### 1.2. Aquaticity as Competence vs. Aquaticity as Adaptive Capacity

Previous studies have operationalized aquaticity primarily in terms of observable aquatic competencies, including buoyancy control, breathing regulation, propulsion, immersion, and underwater perceptual skills, typically within the context of learning and skill acquisition in water sports [6,7]. In such frameworks, aquaticity is assessed as a composite of motor abilities that support safe and effective participation in aquatic activities.

While this competence-based interpretation provides a valuable foundation for assessing aquatic readiness, it conceptualizes aquaticity at the level of task-specific skill execution rather than as a system-level adaptive capacity. The present framework extends this perspective by proposing aquaticity as a latent dimension of organism–environment interaction, reflecting the degree to which general physiological and motor capacities can be functionally expressed under immersion-related constraints. In this sense, observable aquatic competencies may be understood as manifestations of aquaticity, rather than as its defining components.

## 2. Aquaticity in the Existing Literature and Limitations of Previous Definitions

Beyond its etymological origin, the concept of aquaticity has also been discussed in the scientific and professional literature, although relatively infrequently and without a unified meaning. It is most commonly employed as a descriptive expression intended to characterize the general adaptation of the human organism to immersion and movement in an aquatic environment. Its meaning varies depending on the field of application, including rehabilitation, non-swimmer education, water safety, and various forms of recreational and competitive aquatic activities. Such terminological inconsistency has resulted in aquaticity not yet being established as a clearly defined scientific construct within kinesiology and sport science.

The most significant attempt at a systematic scientific examination of the concept of aquaticity can be found in the previous work [6,7]. In these studies, aquaticity is defined as the ability of a terrestrial mammal to function and adapt to the aquatic environment, based on the assumption that cognitive and perceptual–motor adaptation represents the key mechanism of this process. On the basis of this approach, the Aquaticity Assessment Test (AAT) was developed, consisting of a series of psychomotor tasks designed to assess perceptual, coordinative, and cognitive aspects of functioning in water.

The results of these studies demonstrated statistically significant differences in the levels of aquaticity defined in this manner between participants engaged in land-based sports and those involved in aquatic sports, providing important evidence in support of aquaticity as a measurable dimension [6,7]. Furthermore, the authors reported indications of a shared factorial structure among the measured variables, suggesting the possible existence of a latent construct integrating multiple manifest indicators of adaptation to the aquatic environment [6]. These findings represent an important foundation for further conceptual and methodological development in this field.

However, despite the pioneering nature of the aforementioned studies, their applicability within the context of aquatic sports remains limited. First, the definition of aquaticity proposed by Varveri, Karatzaferi, Pollatou and Sakkas [7] primarily focuses on general functioning in water, without explicitly linking the construct to the specific demands of sports performance. In particular, biomechanical factors such as hydrodynamic efficiency, movement economy, and neuromuscular synchronization, key determinants of performance in aquatic sports, are not sufficiently integrated into their conceptual framework [4,8]. Furthermore, physiological adaptations, including respiratory and cardiovascular regulation, autonomic responses, and tolerance to hypoxic–hypercapnic conditions, are not systematically incorporated, despite their well-documented importance in many aquatic sports [9,10,11].

Another major limitation of existing definitions of aquaticity concerns the absence of a discipline-specific perspective. Aquatic sports encompass a wide range of activities with substantially different performance demands, ranging from cyclic movement patterns in swimming, through explosive and anaerobic efforts in finswimming, to prolonged breath-hold and pronounced hypoxic/hypercapniac stress in breath-hold diving. Numerous studies have demonstrated that identical physiological capacities may manifest differently depending on hydrodynamic conditions, movement technique, and discipline-specific breathing constraints [3,12]. Consequently, a universal interpretation of aquaticity, independent of sport discipline, cannot fully account for the variability of performance outcomes observed in aquatic environments.

Within the broader context of sport science, similar conceptual challenges have been recognized in other domains, where latent constructs integrating multiple physiological, biomechanical, and perceptual dimensions of performance are increasingly being adopted [13,14,15]. Such approaches emphasize that sports performance cannot be reduced to the sum of isolated abilities, but rather emerges from their interaction under specific environmental constraints. In this regard, water represents a particularly powerful constraint, profoundly altering the relationships between force production, movement patterns, energetic cost, and perceptual information, thereby further justifying the need for an integrative concept such as aquaticity.

For these reasons, existing definitions of aquaticity, although valuable as an initial step, do not fully meet the needs of aquatic sport kinesiology and applied sport science. The lack of integration between biomechanical, energetic, physiological, and discipline-specific aspects of performance limits their diagnostic and training applicability. This clearly indicates the need for a new theoretical framework in which aquaticity is conceptualized as a latent, multidimensional dimension of sports performance, whose structure can be empirically investigated and operationalized through discipline-specific manifest indicators across different aquatic sports.

## 3. Aquaticity as a Latent Dimension of Sports Performance

In contemporary kinesiology and sport science, there is an increasing need for concepts that transcend the reductionist interpretation of sports performance based on isolated motor, physiological, or technical variables. Numerous authors emphasize that sports performance appears as an emergent property of the interaction between the organism, the task, and the environment, in which individual capacities do not act linearly or additively, but are mutually interdependent and strongly conditioned by the performance context [3,13,14]. This perspective is particularly relevant in sports where the environment profoundly modifies biomechanical and physiological principles of movement, which is especially evident in aquatic environments.

Water as an environment introduces specific constraints that fundamentally alter the relationships between force production, movement, and energetic cost. Hydrodynamic resistance, buoyancy, turbulence, and altered gravitational loading directly affect movement biomechanics while simultaneously inducing changes in respiratory and cardiovascular regulation, blood flow distribution, and sensory perception [16,17,18,19]. In disciplines such as breath-hold diving, complete apnea additionally induces pronounced hypoxic–hypercapnic stress and strong activation of the autonomic nervous system [10,20,21,22,23]. Under such conditions, it becomes evident that aquatic sports performance cannot be explained through a simple extrapolation of land-based measurements or isolated physiological indicators.

Within this context, the present paper proposes the concept of aquaticity as a latent, multidimensional determinant of sports performance specific to the aquatic environment. Aquaticity is not conceptualized as a single ability, skill, or physiological capacity, but as an integrative latent construct reflecting the efficiency and stability of organism–environment interaction in aquatic conditions. As a latent dimension, aquaticity cannot be directly measured by a single variable; instead, it manifests through a set of interrelated performance indicators that reflect how athletes utilize their general capacities under aquatic constraints.

From a theoretical perspective, aquaticity may be understood as a regulatory framework that modulates the transfer of physiological and motor potential into actual performance in water. In other words, it explains why athletes with comparable values of maximal oxygen uptake, muscular strength, or anaerobic capacity may achieve substantially different results in aquatic disciplines. This conceptual approach is consistent with previous findings demonstrating a weak association between land-based physiological tests and performance outcomes in swimming and other aquatic sports, particularly when technical efficiency and movement economy are not taken into account [4,12].

Based on the analysis of existing literature and empirical insights from aquatic sports, aquaticity is herein conceptualized as a construct composed of multiple interrelated domains. These domains do not contribute equally to performance; rather, they are hierarchically and functionally organized, and their relative importance depends on the specific sport discipline. In the broadest sense, the following core components of aquaticity can be identified: biomechanical efficiency of movement in water, energetic optimization, physiological regulation, neurophysiological control, perceptual–mental stability, and discipline-specific technical execution.

The biomechanical component of aquaticity refers to the athlete’s ability to optimize the relationship between force production and hydrodynamic resistance, thereby achieving economical and stable movement in water. It includes effective body positioning, reduction in non-productive drag, coordinated activation of agonists and synergists, and the ability to maintain technical execution under conditions of increased load or fatigue [17,24].

The physiological component encompasses the capacity to regulate respiratory, cardiovascular, and metabolic processes in the aquatic environment. In apnea-based sports, this includes tolerance to hypoxia and hypercapnia, efficiency of autonomic regulation, and the ability to activate the diving response, whereas in sports with continuous breathing, emphasis is placed on economical oxygen utilization and maintenance of stable ventilation under hydrodynamic constraints [10,16,19].

The neurophysiological and perceptual–mental component refers to movement control, stability of reflexive and autonomic responses, and the ability to maintain attention, calmness, and spatial orientation under conditions of altered sensory input and increased physiological stress. Numerous studies indicate a strong association between perceptual–motor control and efficiency of movement in water, particularly under complex or extreme performance conditions [22,25].

In addition to physiological, biomechanical, neurophysiological, and perceptual–mental domains, aquaticity in certain aquatic disciplines includes a distinct functional component related to energy management, herein referred to as energetic aquaticity.

### 3.1. Theoretical Framework of Aquaticity Domains

#### 3.1.1. Physiological Regulation and Neurophysiological Control

The ability to maintain functional homeostasis and performance stability in the aquatic environment through cardiorespiratory and metabolic regulation, including tolerance to hypoxia and hypercapnia and the efficiency of autonomic responses [10,16,19,22,26]. The capacity for fine regulation of autonomic and motor responses (e.g., modulation of sympathovagal balance, neuromuscular coordination, and inhibition of maladaptive reflex responses) under conditions of aquatic stress [13,22,25].

#### 3.1.2. Energetic Aquaticity as Constraint-Governed Resource Deployment

The functional ability to optimally accumulate, distribute, and utilize aerobic–anaerobic energy resources during aquatic performance, particularly under apnea conditions, while preserving critical physiological functions and avoiding premature performance destabilization [10,12,16].

Within the proposed framework, energetic aquaticity should not be interpreted as synonymous with traditional concepts of movement economy or metabolic efficiency, which typically refer to the energetic cost required to sustain a given external workload under conditions of continuous ventilation. In aquatic environments, and particularly in apnea-based disciplines, performance is constrained not only by the total amount of available metabolic energy but also by the temporally and spatially regulated deployment of limited oxygen reserves under conditions of restricted ventilatory compensation.

Energetic aquaticity therefore reflects the capacity to dynamically allocate aerobic and anaerobic metabolic resources in a manner that preserves the critical partial pressure of oxygen required for central nervous system function, while sustaining task-relevant propulsion. This includes pacing strategies that balance peripheral muscular energy expenditure against cerebral oxygen availability, as well as the ability to tolerate localized anaerobic metabolism in propulsion-related musculature without inducing premature systemic destabilization.

In this sense, energetic aquaticity extends beyond conventional notions of efficiency by incorporating regulatory processes that govern metabolic prioritization and oxygen distribution under aquatic constraints. Two athletes with comparable maximal oxygen uptake or movement economy may therefore exhibit markedly different performance outcomes in breath-hold tasks, depending on their ability to preserve functional cerebral oxygenation through effective energetic deployment. Energetic aquaticity thus represents a domain-specific adaptive capacity emerging from the interaction between metabolic regulation, autonomic control, and biomechanical execution under conditions of respiratory limitation.

#### 3.1.3. Biomechanical Efficiency and Perceptual–Mental Stability

The ability to achieve and maintain a hydrodynamically favorable body position and effective propulsion with minimal hydrodynamic resistance and minimal energetic cost of movement [17,18,24].

The ability to maintain attention, calmness, motivational control, and regulation of breathing urge, together with stable perceptual orientation and decision-making under conditions of physiological stress [13,22].

It is important to emphasize that aquaticity cannot be reduced to the sum of these individual components. Rather, it emerges as an emergent property of their mutual interaction, whereby changes within a single domain may exert a disproportionately large effect on overall performance. This nonlinear nature of aquaticity explains why minor technical or perceptual errors in water frequently result in substantial increases in energetic cost or disruption of physiological stability [3,13,14].

By conceptualizing aquaticity as a latent dimension of sports performance, a theoretical framework is established that enables the integration of energetic, biomechanical, physiological, and perceptual aspects into a unified analytical construct. Such a framework not only provides deeper insight into performance in aquatic sports but also creates the basis for the development of new measurement instruments, validation of existing tests, and applied diagnostics and training planning, which will be elaborated in subsequent sections of this paper [13,14].

The conceptual relationship between the latent dimension of aquaticity, its core domains, and sports performance in the aquatic environment is summarized in Table 1.

**Figure 1:**
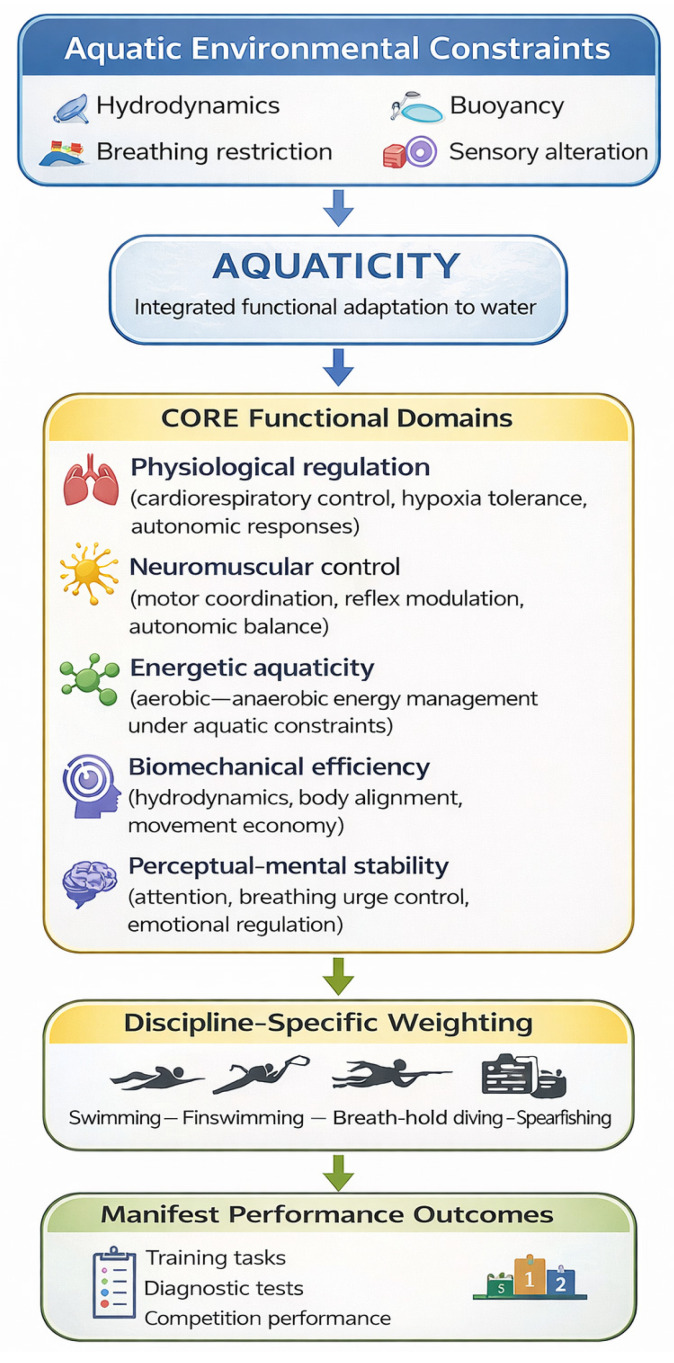
Conceptual framework of aquaticity as an integrative latent dimension of aquatic sports performance.

While Table 1 summarizes the structural components and functional roles of aquaticity, Figure 1 provides an integrative conceptual overview illustrating how aquatic environmental constraints interact with core functional domains to shape sport-specific performance outcomes.

Aquaticity is presented as a multidimensional construct emerging from the interaction between aquatic environmental constraints and key functional domains, including physiological regulation, neuromuscular control, energetic aquaticity, biomechanical efficiency, and perceptual–mental stability. The relative contribution of each domain is discipline-specific and task-dependent. Manifest performance outcomes observed in training, diagnostic tasks, and competition represent functional expressions of aquaticity and serve as ecologically valid indicators for athlete monitoring and performance assessment.

#### 3.1.4. Conceptual Distinction Between Aquaticity and Technical Proficiency

Although aquaticity manifests functionally through observable performance outcomes, it should not be interpreted as synonymous with technical proficiency or sport-specific motor skill. Technical execution refers to the athlete’s ability to produce a task-specific movement solution under stable conditions, whereas aquaticity represents the system-level capacity to preserve the functional integrity of this solution under dynamically interacting aquatic constraints, including hydrodynamic resistance, altered sensory input, and, in certain disciplines, respiratory limitation. In this sense, aquaticity operates as a latent adaptive dimension that modulates the stability, economy, and physiological cost of technical execution rather than the technical solution itself.

Two athletes may therefore exhibit comparable stroke mechanics or propulsion patterns in controlled conditions, yet demonstrate markedly different levels of performance degradation, energetic expenditure, or physiological destabilization when exposed to constraint-amplifying situations such as apnea, fatigue, or repeated underwater efforts. These differences cannot be sufficiently explained by technique alone, but instead reflect the quality of integration between biomechanical, physiological, neuroregulatory, and perceptual–mental processes that collectively determine the robustness of performance under aquatic stress. Within this framework, aquaticity is conceptualized as the adaptive property of the organism–environment system that governs how effectively general physiological and motor capacities are functionally expressed in water.

Accordingly, performance in aquatic sports is not used to define aquaticity per se, but rather to infer its level through the stability and efficiency with which task execution is maintained across varying environmental and physiological constraints. This distinction resolves the potential tautological interpretation of aquaticity and establishes it as a latent construct whose influence is expressed through, but not reducible to, observable technical performance.

## 4. Physiological and Biomechanical Foundations of Aquaticity

Sports performance in aquatic environments is characterized by a specific interaction between biomechanical and physiological factors that differ substantially from those present under terrestrial conditions. Water as a medium introduces increased resistance to movement, altered force interactions, a different distribution of mechanical loading across body segments, and modified sensory input, all of which directly affect movement patterns and energetic demands of performance [16,17,18]. In this context, aquaticity can be understood as a functional framework that enables effective integration of biomechanical and physiological adaptations necessary for stable and economical performance in water.

### 4.1. Biomechanical Efficiency and Hydrodynamic Adaptation

The biomechanical aspects of aquaticity are primarily determined by the relationship between force production and hydrodynamic resistance. Unlike terrestrial locomotion, where movement is largely dominated by overcoming gravity and inertia, movement in water is governed by the continuous need to overcome fluid resistance, which increases exponentially with velocity [4]. Consequently, even minor technical irregularities may lead to a substantial increase in energetic cost and a marked reduction in performance efficiency.

Research in swimming and other aquatic sports consistently demonstrates that propulsive (Froude) efficiency, defined as the ratio of useful mechanical power output to total power expenditure in overcoming hydrodynamic resistance [17,18,24]. This efficiency depends on the athlete’s ability to optimize body position, reduce frontal and turbulent drag, and coordinate the activation of agonists and synergists during propulsion [27]. Within the framework of aquaticity, the biomechanical component encompasses not only the technical execution of movement but also the ability to maintain technical stability under conditions of increased intensity, fatigue, or altered environmental constraints.

In sports involving underwater locomotion, such as breath-hold diving or finswimming, an additional biomechanical challenge arises from the need to maintain body stability in the absence of breathing-related movements and with minimal corrective actions. These conditions require a high level of neuromuscular synchronization and fine regulation of muscle tone, which are directly related to the athlete’s level of aquaticity. Empirical findings indicate that athletes with higher levels of technical and hydrodynamic adaptation exhibit smaller decrements in velocity and greater performance stability during repeated aquatic efforts [12].

### 4.2. Physiological Regulation in the Aquatic Environment

Physiological adaptations in water represent the second fundamental pillar of aquaticity. Immersion and movement in water induce changes in blood distribution, increased venous return, altered ventilatory mechanics, and specific autonomic responses, resulting in cardiovascular and respiratory regulation patterns that differ from those observed under terrestrial conditions [2,28]. These changes may exert a positive effect on the work economy, but only if the athlete is adequately adapted to the aquatic environment.

In disciplines involving continuous breathing, such as swimming, emphasis is placed on the ability to maintain stable ventilation with minimal disruption of biomechanical movement efficiency. Synchronization of breathing and movement, as well as the ability to limit unnecessary respiratory motions, significantly influence the energetic cost of performance [29,30,31]. In this regard, aquaticity also encompasses functional adaptation of the respiratory system to the hydrodynamic demands of movement.

In apnea-based sports, physiological demands are further intensified. Breath-hold diving is characterized by a combination of hypoxia and hypercapnia, accompanied by pronounced activation of the autonomic nervous system and the diving response [22]. Under such conditions, performance in water does not depend solely on absolute physiological capacity, but rather on the ability to regulate and utilize that capacity economically. Athletes with higher levels of aquaticity demonstrate specific altered simultaneous activation of the sympathetic and parasympathetic nervous systems [32,33,34] resulting in more pronounced bradycardia, increased reliance on anaerobic metabolism, more efficient task-specific redistribution of blood flow aimed at enhancing cerebral perfusion, and lower oxygen consumption for the same mechanical output. These adaptations enable longer and safer execution of apnea-based tasks.

### 4.3. Interaction Between Biomechanics and Physiology

A key characteristic of aquaticity is the strong interdependence between biomechanical and physiological factors. Unlike terrestrial sports, where technical errors can often be partially compensated by increased energetic expenditure, such a strategy in water leads to a disproportionately large rise in energetic cost and accelerated physiological destabilization. Research has shown that even minimal increases in hydrodynamic resistance result in significant elevations in oxygen consumption and accumulation of metabolic by-products [4].

This nonlinear interaction between biomechanics and physiology renders the aquatic environment particularly sensitive to the athlete’s level of aquaticity. In practical terms, this means that technical stability, movement economy, and perceptual control have direct physiological consequences, especially under conditions of high intensity or restricted breathing. This relationship further supports the conceptualization of aquaticity as an integrated dimension of sports performance, rather than as a collection of isolated biomechanical or physiological variables.

### 4.4. Implications for Understanding Aquatic Sports Performance

Understanding the physiological and biomechanical foundations of aquaticity enables a deeper explanation of performance variability in aquatic sports. Rather than attributing success exclusively to levels of strength, endurance, or aerobic capacity, aquaticity provides a framework for understanding how these capacities are functionally realized under the specific constraints of the aquatic environment. This perspective establishes a theoretical basis for the development of measurement instruments and training protocols that account for the integrated nature of biomechanical and physiological adaptations.

This chapter lays the groundwork for further elaboration of discipline-specific aspects of aquaticity and its application in individual aquatic sports, which will be addressed in the following sections through a general overview of aquatic sports and a detailed analysis of breath-hold diving as a model of applied aquaticity.

## 5. Aquaticity in Aquatic Sports: General and Discipline-Specific Aspects

Aquatic sports encompass a wide range of disciplines that, despite sharing a common environment, differ substantially in biomechanical movement patterns, energetic demands, breathing modalities, and the degree of exposure to physiological stress. Swimming, finswimming, breath-hold diving, and spearfishing represent only some examples of sports in which water acts as the dominant environmental constraint, while performance is realized through different combinations of technical, physiological, and perceptual–mental demands. This diversity further emphasizes the need for an integrative concept capable of explaining both shared and discipline-specific elements of adaptation to the aquatic environment.

In this context, aquaticity can be viewed as a common foundational dimension underlying all aquatic sports, while exhibiting discipline-specific patterns of manifestation. In other words, although the primary function of aquaticity is to enable efficient interaction between the organism and the aquatic medium, the relative contribution of individual aquaticity domains differs depending on the specific demands of a given sport discipline. This perspective is consistent with contemporary models of sports performance that conceptualize performance as the outcome of interactions among the organism, the task, and the environment, whereby changes in any of these elements alter the hierarchy of relevant capacities [13,14].

In swimming, as a cyclic activity with continuous breathing, the biomechanical component of aquaticity plays a particularly dominant role. Hydrodynamic efficiency, movement economy, and technical stability during prolonged workloads are key determinants of success, whereas physiological capacities such as aerobic endurance can only be effectively expressed if they are adequately integrated into an efficient technical movement pattern [4,17]. Numerous studies have demonstrated that differences in technique and hydrodynamic drag can explain a substantial proportion of variance in swimming performance, independently of laboratory-measured physiological parameters [12].

In finswimming and related disciplines, the role of neuromuscular synchronization and discipline-specific propulsion biomechanics is further emphasized. The use of fins alters force–resistance relationships, increases swimming velocity, and imposes greater demands on trunk stability and control of movement amplitude. In these disciplines, aquaticity manifests through the athlete’s ability to exploit the mechanical advantages of equipment without compromising hydrodynamic efficiency or physiological stability, particularly at high exercise intensities.

In contrast to apnea-based disciplines that amplify physiological constraints through ventilatory limitation, artistic swimming represents a performance environment in which multiple aquatic demands are simultaneously integrated. Athletes are required to coordinate propulsion, buoyancy control, spatial orientation, and respiratory timing under conditions of intermittent immersion, often while maintaining synchronization with external auditory stimuli and complex movement sequences. These demands necessitate the preservation of perceptual–motor stability and movement precision despite transient hypoxic exposure and altered vestibular input.

Within this context, artistic swimming provides an ecologically valid example of how aquaticity may manifest beyond extreme apnea-based performance, through the integration of biomechanical, physiological, and perceptual–mental processes required for coordinated action in water. As such, it may be considered a representative multidomain expression of aquatic adaptation, complementing the constraint-amplifying role of breath-hold diving within the broader conceptual framework proposed in the present study.

In addition to individual disciplines, team-based aquatic sports such as water polo require the maintenance of propulsion, vertical support, spatial awareness, and tactical decision-making under conditions of intermittent immersion and constrained breathing. Athletes must repeatedly transition between submerged and surface actions while executing task-relevant movements in dynamic and contact-rich environments. These demands impose not only biomechanical and physiological challenges, but also perceptual–cognitive constraints related to decision latency and movement selection under immersion.

As such, water polo provides an example of how aquaticity may influence performance stability in interactive and tactically complex aquatic settings, where effective movement execution depends on the coordinated integration of motor, autonomic, and perceptual–mental processes.

In contrast to the aforementioned disciplines, breath-hold diving represents an extreme example of an aquatic sport in which the physiological component of aquaticity becomes especially prominent. Complete apnea, hypoxic–hypercapnic stress, and activation of the diving response create conditions in which biomechanical efficiency, mental control, and autonomic regulation cannot be considered independently. Research in breath-hold diving indicates that performance is not determined solely by lung capacity or maximal physiological potential, but rather by the ability to stably regulate heart rate, oxygen consumption, and psychological calmness during exertion [22].

Spearfishing, although often underrepresented in scientific literature, further emphasizes the perceptual–mental and safety-adaptive components of aquaticity. Unlike competitive freediving, performance in spearfishing involves real-time decision-making, hazard recognition, risk assessment, spatial orientation in a variable environment, and adaptation to unpredictable situations. Under such conditions, aquaticity is expressed not only through technical and physiological efficiency, but also through the ability to maintain control and safety in complex environmental contexts.

It is important to emphasize that aquaticity cannot be universally quantified using identical measurement instruments across all aquatic sports. Although the core domains of aquaticity remain consistent, their relative importance and modes of operationalization must be adapted to the specific demands of each discipline. This discipline-specific approach distinguishes the concept of aquaticity from earlier, more generalized attempts to describe adaptation to the aquatic environment and renders it applicable within both scientific diagnostics and training practice.

By examining aquaticity through the lens of different aquatic sports, a theoretical foundation is established for its further operationalization and empirical validation. At the same time, this approach prepares the ground for a more detailed analysis of breath-hold diving as a sport in which environmental constraints, physiological stress, and safety considerations are particularly pronounced. For this reason, breath-hold diving is used in the following sections as a model for illustrating applied aquaticity and its diagnostic and training relevance.

## 6. Breath-Hold Diving as a Model of Applied Aquaticity

Breath-hold diving represents a specific and methodologically highly valuable model for the study of aquaticity, as this sport discipline expresses, in an extreme manner, the interaction between biomechanical, metabolic, physiological, neurophysiological, and perceptual–mental factors under conditions of strong environmental constraint. Unlike most aquatic sports in which breathing is continuous or only partially restricted, breath-hold diving is characterized by complete apnea, whereby performance is directly conditioned by hypoxic–hypercapnic stress, activation of the autonomic nervous system, and strictly limited energetic resources [22,35].

Under such conditions, the athlete’s ability to effectively realize physiological and motor potential in water depends to a far greater extent on the level of aquaticity than on absolute values of individual capacities. Breath-hold diving therefore acts as a form of “amplifier” of latent differences in adaptation to the aquatic environment, rendering aquaticity a particularly visible and measurable dimension of sports performance.

### 6.1. Breath-Hold Diving as a Constraint-Amplified Model of Aquatic Adaptation

It is important to emphasize that breath-hold diving is not presented in this paper as a representative model of aquatic sports performance in general, but rather as a constraint-amplified performance environment in which latent differences in adaptation to the aquatic medium become particularly pronounced. Unlike disciplines characterized by continuous breathing, apnea-based performance imposes strict limitations on ventilatory compensation, thereby increasing the sensitivity of performance outcomes to biomechanical inefficiencies, suboptimal pacing strategies, or instability in autonomic and perceptual regulation. In this context, breath-hold diving functions as a methodological “stress test” of the organism–environment interaction in water, allowing subtle variations in the integration of physiological, biomechanical, and perceptual–mental domains to manifest as measurable differences in performance stability, energetic cost, or recovery dynamics.

Accordingly, the role of breath-hold diving within the present conceptual framework is not to serve as a universal proxy for aquatic sports, but to provide an experimentally tractable setting in which the functional expression of aquaticity can be observed under heightened environmental and physiological constraints. This approach is analogous to the use of altitude exposure in terrestrial sport science, where hypobaric or hypoxic conditions are employed not as representative of everyday performance environments, but as constraint-amplifying contexts that reveal underlying adaptive capacities otherwise less detectable under normoxic conditions.

### 6.2. Specific Constraints of Breath-Hold Diving

The fundamental characteristic of breath-hold diving is the execution of motor activity under conditions of complete apnea, thereby eliminating the possibility of immediate compensation for increased energetic demands through enhanced ventilation. Consequently, any biomechanical inefficiency, technical instability, or unnecessary activation of muscle tone has a direct and disproportionate effect on the rate of oxygen consumption and carbon dioxide accumulation.

In addition, breath-hold diving involves pronounced activation of the diving reflex, manifested through bradycardia, peripheral vasoconstriction, and redistribution of blood flow toward vital organs [11]. Although this reflex is fundamentally protective in nature, its effectiveness in a sporting context largely depends on the athlete’s training status and the ability to maintain biomechanical and mental stability under load. Breath-hold diving thus clearly demonstrates that physiological responses alone are insufficient for successful performance unless they are integrated into the overall pattern of aquaticity.

The perceptual–mental domain of aquaticity in breath-hold diving extends beyond psychological calmness and control of the breathing urge. It also encompasses highly developed aquatic-specific perception and proprioception, including the ability to assess direction and velocity of movement, detect changes in hydrodynamic forces, maintain spatial orientation, and precisely control body position and movement without visual feedback. Such sensorimotor regulation represents a key prerequisite for technical stability, energetic efficiency, and safety of performance in aquatic environments.

### 6.3. Hierarchical Organization of Aquaticity Domains in Breath-Hold Diving

In breath-hold diving, the hierarchical organization of relevant aquaticity domains becomes particularly pronounced. Physiological regulation and neurophysiological control constitute the necessary foundation of performance, as tolerance to hypoxia and hypercapnia sets absolute limits on the duration and intensity of activity. However, these limits become functionally exploitable only when supported by adequate accumulation of aerobic–anaerobic metabolic capacities, a high level of biomechanical efficiency, and perceptual–mental stability.

The metabolic–energetic domain of aquaticity in breath-hold diving refers to the ability to accumulate, preserve, and functionally utilize aerobic–anaerobic energy capacities under conditions of apnea and reduced peripheral perfusion. In breath-hold diving disciplines, activation of the diving reflex leads to pronounced centralization of blood flow and reduced oxygen delivery to peripheral musculature, particularly the muscles responsible for propulsion. As a consequence, these muscles are forced to cover an increasing proportion of their energetic demands through anaerobic pathways, despite relatively low absolute mechanical workload.

In this context, performance in dynamic and depth disciplines does not depend solely on the total amount of available energy, but rather on the strategy of its temporal distribution. Breath-hold divers, through conscious or implicit pacing strategies, attempt to optimize the balance between anaerobic energy expenditure in peripheral musculature and preservation of the critical partial pressure of oxygen required to maintain consciousness. Higher propulsion velocity increases instantaneous energetic demand and reliance on anaerobic metabolism, whereas slower movement prolongs dive duration but increases overall exposure to hypoxia.

Accordingly, metabolic–energetic aquaticity manifests as the athlete’s ability to maximally deplete anaerobic energy stores in peripheral musculature without compromising cerebral oxygenation below a critical threshold. The level of this capacity is directly related to the anaerobic and buffering capacities of propulsion-related muscles, which are developed through a combination of general dry-land conditioning and specific high-intensity and load-based aquatic training.

Within the aquaticity framework, this domain represents a functional link between physiological constraints and biomechanical efficiency, as even an optimal technique loses performance value if the athlete lacks sufficient energetic capacity to maintain it throughout the entire dive.

The biomechanical component of aquaticity in breath-hold diving primarily refers to the ability to maintain a stable, hydrodynamically favorable body position with minimal energetic expenditure. Underwater locomotion, particularly when using a monofin or bifins, requires precise control of movement amplitude and frequency, as well as optimal neuromuscular synchronization. Research and training practice indicate that even small technical deviations under these conditions lead to a rapid increase in energetic cost and accelerated depletion of limited respiratory reserves [25].

The perceptual–mental domain of aquaticity plays an exceptionally important role in breath-hold diving. Control of the breathing urge, maintenance of mental calmness, and the ability to sustain focused attention under acute respiratory stress represent key prerequisites for stable performance. Numerous studies demonstrate a strong association between psychological regulation, autonomic responses, and the effectiveness of the diving reflex, further confirming that the mental component is not separate from physiology, but rather an integral part of it [22].

The hierarchical organization of aquaticity domains in breath-hold diving is summarized in Table 2.

### 6.4. Aquaticity as an Explanation of Performance Variability in Freediving

One of the key practical values of the aquaticity concept in breath-hold diving lies in its ability to explain pronounced performance differences between athletes with formally similar physiological capacities. In training and competitive practice, it is common to observe situations in which athletes with comparable lung volumes, maximal oxygen uptake, or anaerobic capacity achieve substantially different results in static and dynamic apnea.

The concept of aquaticity enables interpretation of these differences through the quality of integration between physiological, energetic, biomechanical, and mental components. Athletes with a higher level of aquaticity are able to maintain technical stability, minimal unnecessary muscle tone, and efficient autonomic regulation throughout the entire performance, thereby reducing the rate of oxygen consumption and prolonging tolerance to respiratory stress [4,22]. In contrast, athletes with lower aquaticity levels tend to exhibit earlier technical destabilization, increased non-functional muscle activation, and accelerated physiological decompensation.

Precisely because of these characteristics, breath-hold diving represents an exceptionally suitable model for studying aquaticity as a latent dimension of sports performance. The extreme conditions of apnea, clearly defined safety limits, and high sensitivity of performance to technical and perceptual changes allow for relatively clear identification of manifest indicators of aquaticity. These indicators, operationalized through specific training and diagnostic elements, are addressed in greater detail in the following chapter.

## 7. Training and Diagnostic Elements as Empirical Indicators of Aquaticity

Conceptualizing aquaticity as a latent dimension of sports performance requires appropriate manifest indicators through which it can be empirically observed, monitored, and interpreted. In aquatic sports and particularly in breath-hold diving, such indicators rarely take the form of isolated laboratory measurements. Instead, they most often emerge through the quality of task execution in real aquatic environments, where biomechanical, energetic, physiological, and perceptual–mental components of aquaticity are simultaneously engaged [6,7]. Development of portable telemonitoring physiological devices worn by breath-hold divers is necessary to supplement the intense and extreme nature of this sport.

It is important to distinguish between the latent capacity represented by aquaticity and the situational factors that may influence its observable expression during performance in aquatic environments. External environmental and equipment-related variables, such as water temperature, salinity, buoyancy characteristics, wetsuit thickness, or fin stiffness, may significantly alter the mechanical and physiological demands of aquatic activity by modifying hydrodynamic resistance, thermal stress, or propulsion efficiency.

However, these factors do not constitute components of aquaticity itself, but rather act as modulators of the conditions under which aquaticity is functionally expressed. For example, increased buoyancy resulting from wetsuit use may reduce the energetic cost of maintaining body position in water, while fin-assisted propulsion may alter force–resistance relationships and movement coordination patterns. Such modifications influence performance outcomes without necessarily reflecting a change in the underlying level of organism–environment adaptation.

Within this framework, aquaticity is interpreted as a latent adaptive capacity of the athlete, whereas environmental and equipment-related variables determine the boundary conditions under which this capacity becomes observable. This distinction allows performance variability arising from situational factors to be analytically separated from differences in aquaticity itself, thereby preserving the conceptual integrity of the construct across diverse aquatic contexts.

In this context, the present paper introduces the concept of training elements as dual-purpose tasks that function both as training stimuli and as diagnostic tools. These elements are characterized by clearly defined motor tasks, standardized execution conditions, and quantifiable performance outcomes, enabling their use in longitudinal monitoring of athletes across different levels of training status.

From an applied perspective, aquaticity-based training and diagnostic elements may serve as ecologically valid tools for athlete monitoring, capturing functional adaptations that are not detectable through isolated laboratory measurements. Within this framework, several commonly used training and diagnostic practices in breath-hold diving can be interpreted as observable manifestations of the underlying latent dimension of aquaticity.

### 7.1. Training Elements as Manifest Variables of a Latent Dimension

Several training and diagnostic elements commonly used in breath-hold diving can be interpreted as indirect indicators of energetic aquaticity, as they challenge the athlete’s ability to manage limited energy resources under progressive hypoxic and hypercapnic stress [8,9].

Unlike classical physiological tests that isolate individual capacities such as maximal oxygen uptake or blood lactate response, training elements performed in aquatic environments reflect how these capacities are functionally realized under real performance conditions. As such, they do not directly measure aquaticity itself, but rather serve as manifest variables that reflect its level [10].

A key advantage of this approach is its high ecological validity. Because tasks are performed in real aquatic environments under authentic biomechanical and physiological constraints, the outcomes of training elements are directly related to actual sports performance and safety-related aspects of activity. In this way, aquaticity is operationalized in a manner that is both scientifically grounded and practically applicable [12].

### 7.2. Swimming Anaerobic Sprint Test (SAST)

The Swimming Anaerobic Sprint Test (SAST) was developed to assess sport-specific anaerobic sprint endurance in swimming. The test consists of repeated short-distance bouts performed at maximal or submaximal intensity under strictly controlled recovery conditions. Although SAST is primarily designed to evaluate anaerobic capacity, its outcomes are strongly influenced by the athlete’s level of technical proficiency and hydrodynamic adaptation to the aquatic environment [13,14,36].

The use of SAST in assessing anaerobic capacities relevant to breath-hold diving has been supported by empirical studies demonstrating significant changes in sprint-test performance following targeted training interventions [36].

Within the aquaticity framework, SAST can be interpreted as an indirect indicator of biomechanical and perceptual–motor adaptation to the aquatic environment. Athletes with higher levels of aquaticity typically exhibit smaller velocity decrements between repetitions, greater technical stability, and lower energetic cost of movement at comparable external workloads [29,31]. In contrast, athletes with lower aquaticity levels tend to demonstrate earlier technical degradation, increased hydrodynamic resistance, and faster onset of fatigue, despite formally similar physiological capacities [28].

### 7.3. Diving Anaerobic Sprint Test (DAST)

The Diving Anaerobic Sprint Test (DAST) represents a sport-specific adaptation of anaerobic sprint testing for conditions of complete breath-hold. In contrast to SAST, DAST is performed during underwater locomotion without breathing, thereby further emphasizing the integration of physiological, biomechanical, and mental components of aquaticity.

DAST reflects the athlete’s ability to express anaerobic potential under hypoxic–hypercapnic stress, while simultaneously maintaining technical stability and mental control, in accordance with previously described physiological and neurophysiological mechanisms of breath-hold diving [35,36]. Test outcomes are strongly dependent on the regulation of autonomic responses, the economy of underwater movement, and the control of the breathing urge. Consequently, DAST represents an exceptionally sensitive indicator of aquaticity level, particularly within trained breath-hold diver populations. Different BHD disciplines (static, dynamic, and spearfishing) affect different metabolic/energy sources [37]. Nutritional requirements differ between freediving (static and dynamic) and spearfishing, mainly because of the workload.

Hence, the nutrition of these athletes may play an important role in their performance. A Mediterranean and alkaline diet could represent an appropriate BHD diet.

### 7.4. Prolonged and Combined Training Elements

In addition to sprint-based tests, longer-duration and combined tasks are commonly used in training and diagnostic practice to assess the temporal stability of aquaticity. Examples of such elements include repeated apnea-based efforts (e.g., 16 × 50 m breath-hold dives), prolonged endurance tests (e.g., a 12-minute Cooper test performed in swimming or breath-hold diving), and higher-volume underwater swimming series (e.g., 10 × 100 m in apnea), all performed with minimal recovery intervals.

In these tasks, aquaticity manifests through the athlete’s ability to maintain stable performance, regulate pace, minimize unnecessary muscular tension, and efficiently distribute limited energetic resources over time. Particularly informative indicators include total task completion time, velocity variability between repetitions, duration of required recovery, and the preservation of technical quality during later phases of the task.

### 7.5. Reference Performance Profiles and Levels of Aquaticity

The following description is based on long-term empirical observations obtained from training practice with competitive breath-hold divers and should be interpreted as a qualitative synthesis of recurring performance patterns, rather than as evidence derived from a formally designed experimental study.

The application of the above-mentioned training and diagnostic elements in long-term practice has enabled the formation of reference performance profiles corresponding to different levels of training status, including beginners, recreational divers, competitive athletes, and elite breath-hold divers. Although detailed normative values are not presented in this paper, clear and consistent quantitative and qualitative differences in performance patterns can be observed between these groups.

Elite athletes are characterized by high technical stability, minimal performance decrement across repeated efforts, and a pronounced ability to regulate physiological and mental responses to progressive hypoxic–hypercapnic stress. Such athletes typically exhibit higher levels of discipline-specific aerobic and anaerobic capacities, enhanced buffering capacity of the peripheral muscles involved in propulsion, and more effective redistribution of blood flow during apnea. Together, these adaptations support the preservation of cerebral perfusion and maintenance of consciousness stability during demanding tasks [38,39,40].

From a physiological perspective, higher levels of aquaticity are associated with a more pronounced and rapidly activated diving response, including bradycardia, peripheral vasoconstriction, and centralization of blood flow [2,41,42,43,44,45,46]. These mechanisms, in combination with increased vascular compliance of cerebral blood vessels, enable more effective regulation of cerebral oxygenation and accelerated carbon dioxide wash-out from the cerebral circulation. Consequently, athletes with higher aquaticity levels demonstrate greater physiological and mental resistance to cerebral hypoxia and asphyxia.

In practical terms, one of the most clearly observable indicators of higher aquaticity is the ability to shorten recovery intervals during successive repetitions of short- and medium-duration breath-hold dives without compromising technical quality or safety. This phenomenon suggests reduced chemoreceptor sensitivity to elevated CO_2_ levels, as well as more efficient integration of autonomic and vascular compensatory mechanisms developed through specific training stimuli [47].

In contrast, less trained and less experienced divers typically exhibit greater performance variability, faster technical degradation, prolonged recovery requirements between repetitions, and more pronounced physiological instability. Such patterns indicate a lower degree of integration across the relevant domains and further confirm that training elements used in practice function as valid manifest indicators of the latent dimension of aquaticity, particularly in disciplines characterized by repeated dives, such as breath-hold diving and spearfishing.

Beyond standardized training and diagnostic elements, maximal performance in specific competitive breath-hold diving disciplines may also be considered a discipline-specific and comprehensive indicator of aquaticity level. Each freediving discipline possesses its own hierarchical structure of performance determinants, in which individual aquaticity domains are weighted differently according to the biomechanical, physiological, and perceptual–mental demands of the discipline.

In this context, an athlete specialized in a given discipline typically demonstrates a higher level of aquaticity within that discipline compared to others, reflecting the degree of specific adaptation to its dominant constraints. Maximal competitive performance therefore represents not merely the final outcome of the training process but may be interpreted as a synergistic resultant of manifest variables, and thus as the closest practical approximation of the latent aquaticity space under real performance conditions.

### 7.6. Methodological Implications

Viewing training and diagnostic elements as empirical indicators of aquaticity enables the establishment of a methodological bridge between the theoretical framework of a latent dimension and its practical application in real aquatic environments. Rather than reducing a complex and multidimensional phenomenon to a single isolated test, this approach allows for a multivariate assessment of adaptation to the aquatic environment, while preserving high ecological validity and a direct link to performance and safety-related aspects of activity [6,7].

In the present paper, a limited set of training elements is presented for which empirical data and extensive practical experience suggest sensitivity to the level of aquaticity. However, it is important to emphasize that these elements do not represent a final or exhaustive measurement system. On the contrary, in applied practice there exists a broad range of additional situational training elements that may reflect different aspects of aquaticity, but which still require methodological standardization and validation before they can be incorporated as reliable measurement instruments.

Such an expanded set of manifest variables would enable the application of *factor analysis* and other multivariate statistical methods aimed at identifying the structure of the latent aquaticity space. Grouping individual training elements into common factors would allow for a more precise understanding of which aquaticity domains are predominantly assessed by specific tests, as well as their interrelationships and hierarchical organization within the overall construct [48]. Future research should aim to empirically examine the structure of aquaticity using multivariate statistical approaches (e.g., factor analysis or structural equation modeling) in order to identify the latent relationships between physiological, biomechanical, energetic, and perceptual–mental indicators of aquatic performance. In addition, maximal performance in specific competitive disciplines may be methodologically operationalized as a synthetic indicator of aquaticity through the introduction of a *competition index*, defined as the relative deviation of an achieved result from the current world record or another relevant reference value. The competition index can be formally expressed as CI = P/R, where P represents the athlete’s achieved performance and R the corresponding reference value (e.g., the current world record or another relevant benchmark). Such an index would allow for a quantitative assessment of overall discipline-specific aquaticity under real competitive conditions, with maximal performance interpreted as an integrative outcome of all manifest variables involved in execution.

Similarly, by establishing a database of training and diagnostic results obtained from a large number of athletes across different training levels, it becomes possible to define reference ranges representing the best and weakest performances for individual tasks. Expressing individual results as relative deviations from these reference values enables the construction of a training position index within a specific aquaticity component, thereby facilitating longitudinal monitoring, athlete comparison, and the individualization of training interventions.

Such a multi-level methodological approach opens the way for longitudinal studies of adaptation, the development of discipline-specific diagnostic batteries, and the gradual quantification of the latent dimension of aquaticity, while preserving its theoretical coherence and practical relevance in aquatic sports.

## 8. Implications for Measurement, Training, and Safety in Aquatic Sports

Conceptualizing aquaticity as a latent dimension of sports performance has important implications for how ability assessment, training planning, and safety management are approached in aquatic sports. In contrast to traditional models that interpret performance through isolated physiological or motor parameters, an aquaticity-based framework enables an integrated assessment of the athlete’s adaptation to the specific environmental constraints imposed by water.

### 8.1. Implications for Sports Diagnostics and Performance Measurement

Introducing aquaticity as a theoretical framework highlights the limitations of standard diagnostic procedures that rely exclusively on land-based tests or isolated physiological measurements. Although such tests provide valuable information about an athlete’s general capacity, they often fail to explain performance variability in aquatic environments, particularly in disciplines characterized by pronounced biomechanical and respiratory constraints [7,10].

The use of training elements as diagnostic tools allows aquaticity to be assessed through actual performance outcomes in water. Rather than relying on a single measure, the level of aquaticity becomes evident through performance patterns such as technical stability, movement economy, recovery dynamics, and the ability to maintain control under progressively increasing load. This approach enables the development of multidimensional diagnostic profiles, in which individual indicators are interpreted relationally rather than in isolation.

In the long term, such a conceptual framework may serve as a foundation for the development of standardized, sport-specific test batteries for aquatic disciplines, in which aquaticity is operationalized through a set of validated manifest indicators. This would allow improved comparability of results across athletes, coaches, and researchers, while preserving high ecological validity of measurement procedures.

### 8.2. Implications for Training Planning and Individualization

Within training practice in aquatic sports, the concept of aquaticity enables more precise individualization of the training process. Instead of a uniform approach based solely on training volume and intensity, training interventions can be directed toward specific aquaticity domains that represent performance-limiting factors in a given athlete. This approach is particularly relevant in breath-hold diving, where small deviations in biomechanical efficiency or mental control may have a substantial impact on both performance and safety.

Monitoring longitudinal changes in the performance of training elements provides insight into the adaptive dynamics of aquaticity, rather than merely tracking changes in isolated physiological capacities. In this way, the training process can be aligned with the athlete’s actual level of adaptation to the aquatic environment, thereby reducing the risk of overload and enhancing training effectiveness. Furthermore, this framework allows differentiation between technical, motor, functional–metabolic, physiological, and perceptual limitations, which is essential for the long-term development of high-level athletes in aquatic disciplines.

### 8.3. Implications for Safety in Aquatic Sports

Safety considerations represent one of the most important areas of application of the aquaticity concept, particularly in sports involving apnea and hypoxic stress. Numerous incidents in breath-hold diving and other aquatic activities are associated with inadequate assessment of an athlete’s actual level of adaptation to the aquatic environment, where formal physiological capacities alone do not constitute reliable indicators of execution safety [2].

By viewing aquaticity as an integrated dimension, it becomes possible to identify potential risks at an earlier stage, such as technical instability, excessive autonomic reactivity, or insufficient load regulation under apnea conditions. Training elements, when used as diagnostic tools, may serve as functional safety tests, as they reveal how an athlete responds to progressively increasing stress under controlled conditions.

In this sense, aquaticity should be regarded not only as a determinant of sports performance but also as a critical factor in accident prevention and optimization of safety protocols. Incorporating aquaticity assessment into education systems, athlete selection, and progression frameworks may substantially contribute to risk reduction in aquatic sports, particularly at higher levels of technical and physiological demand.

### 8.4. Perspectives for the Development of Integrated Aquaticity Indices

Although aquaticity is not operationalized in this paper through a single numerical index, the conceptual framework presented here opens the possibility for the future development of such instruments. The integration of biomechanical, energetic, physiological, and perceptual indicators into complex models may enable quantitative assessment of aquaticity levels, while preserving discipline-specific sensitivity.

Such approaches will require interdisciplinary research, longitudinal study designs, and advanced statistical methods. However, the potential benefits in terms of more precise diagnostics, improved training management, and enhanced safety justify such efforts. Within this perspective, aquaticity may be viewed as a platform for the future development of scientific and applied tools in aquatic sports.

As such, aquaticity represents not only a conceptual advance but also a functional kinesiology framework with direct relevance for sport biomechanics, exercise physiology, neuromuscular control, and safety-oriented athlete monitoring in aquatic sports.

## 9. Limitations and Future Research Directions

Despite its clear theoretical grounding and high applied relevance, the present paper has several limitations arising from the very nature of the aquaticity concept and the methodological approach adopted. First, aquaticity is defined and elaborated here as a latent, multidimensional dimension of sports performance, which necessarily implies that it cannot be directly measured using a single isolated test or physiological indicator. The empirical component of this paper is therefore based on the interpretation of training and diagnostic elements as manifest indicators of aquaticity, rather than on a formal statistical validation of the construct.

A second limitation relates to the fact that the empirical examples presented in this paper are predominantly focused on breath-hold diving. While this choice is methodologically justified due to the pronounced sensitivity of freediving and spearfishing to biomechanical, energetic, physiological, and perceptual–mental factors, it simultaneously limits the direct generalization of the findings to all aquatic sports. Nevertheless, the theoretical framework of aquaticity has been intentionally designed to allow discipline-specific operationalization, thereby providing a foundation for future research in swimming, finswimming, and other aquatic disciplines.

Third, this paper does not present normative values or quantitative thresholds for different levels of aquaticity. This decision was made deliberately in order to avoid the premature reduction of a complex construct to simplified numerical indicators. However, the development of such normative frameworks represents an important direction for future research, particularly in the contexts of athlete selection, developmental monitoring, and safety risk assessment.

Future research should therefore be directed toward the empirical validation of aquaticity as a latent construct, including factor-analytic approaches to manifest indicators, longitudinal tracking of adaptive processes, and comparisons across different athlete populations. Particular potential lies in the integration of biomechanical measurements, estimated energetic demands, physiological parameters, and perceptual–mental indicators into complex multivariate models that would allow quantitative assessment of aquaticity levels. Furthermore, future studies should explore whether energetic aquaticity can be operationalized and quantified through standardized in-water performance protocols. Such an interdisciplinary approach may ultimately lead to the development of standardized, yet discipline-specific, diagnostic batteries for aquatic sports.

## 10. Conclusions

In this paper, aquaticity is presented as an integrated, latent, and multidimensional determinant of sports performance specific to the aquatic environment. Building on the limitations of existing approaches that often interpret success in aquatic sports through isolated physiological, technical, or biomechanical variables, a conceptual framework is proposed that integrates biomechanical, energetic, physiological, neurophysiological, and perceptual–mental adaptations into a single analytical entity. This framework enables a deeper understanding of how general athlete capacities are functionally realized in aquatic conditions and explains why athletes with comparable baseline abilities may achieve markedly different levels of performance.

Breath-hold diving is presented as a particularly suitable model for studying aquaticity, as the extreme conditions of apnea and hypoxic–hypercapnic stress clearly expose differences in the degree of adaptation to the aquatic environment. By interpreting training and diagnostic elements as manifest indicators, aquaticity can be operationalized within real aquatic settings while maintaining high ecological validity and a direct relationship with performance and safety-related outcomes. Within this context, aquaticity can be empirically observed through technical stability, regulation of physiological and mental responses, and performance patterns during repeated and maximal tasks.

Within the proposed framework for breath-hold diving, energetic aquaticity is highlighted as a central functional sub-construct linking physiological constraints, biomechanical efficiency, and perceptual–mental stability. Maximal competitive performance in specific freediving disciplines represents a comprehensive manifestation of aquaticity, reflecting a synergistic outcome of multiple manifest variables, and may therefore be considered the closest practical approximation of the latent aquaticity space under real performance conditions.

The concept of aquaticity has important implications for sports diagnostics, training planning, and safety management in aquatic sports. Its application enables a more individualized, effective, and safer approach to athlete development, while simultaneously enhancing understanding of the complex interaction between the human organism and the aquatic environment. Future research should focus on expanding and validating discipline-specific diagnostic batteries, applying multivariate statistical methods to identify the structure of the latent aquaticity space, and developing standardized indices for longitudinal monitoring of adaptations and comparison of athletes across different aquatic sports.

In conclusion, aquaticity is not proposed merely as a new theoretical term, but as a functional scientific framework with clear empirical, methodological, and practical relevance, with the potential to significantly advance kinesiology and sports science in the domain of aquatic sports. Although the present framework is developed primarily within the context of aquatic sports, the conceptual approach may also have broader relevance for understanding performance in other environments where specific environmental constraints strongly modulate the expression of physiological and motor capacities. As a conceptual contribution, this paper provides a theoretical foundation intended to guide future experimental, diagnostic, and applied research in aquatic sports.

## Figures and Tables

**Table 1:** Conceptual framework of aquaticity as a latent dimension of performance in aquatic sports. AQUATICITY is presented as a latent construct emerging from the interaction of physiological regulation, neurophysiological control, energetic aquaticity, biomechanical efficiency, and perceptual–mental stability under the constraints of the aquatic environment. The construct manifests through sport-specific performance and can be indirectly assessed using training and diagnostic elements applied in real aquatic conditions.

Level/Component	Description	Role in Aquaticity
Aquatic environment (constraints)	Hydrodynamic resistance, buoyancy, altered sensory input, breathing constraints	Defines the specific physical and physiological conditions under which aquatic performance is realized
Aquaticity (latent dimension)	Integrated, multidimensional adaptation of the organism to the aquatic environment	Represents the fundamental latent determinant of success in aquatic sports
Physiological regulation	Tolerance to hypoxia and hypercapnia; cardiorespiratory control	Defines the limits of duration and intensity of activity in aquatic conditions
Neurophysiological control	Autonomic regulation and sympathovagal balance; motor coordination	Enables stability of autonomic responses and precise motor control under stress
Energetic aquaticity	Accumulation, distribution, and utilization of aerobic–anaerobic energy resources	Mediates between physiological capacities and actual performance with minimal energetic loss
Biomechanical efficiency	Hydrodynamics, body position stability, minimal energetic cost of movement	Reduces drag and energetic cost of movement in water
Perceptual–mental stability	Aquatic-specific perception and proprioception; breathing urge control; focus and emotional calmness	Enables maintenance of performance under hypoxic–hypercapnic stress
Aquatic sports performance	Maximal or repeated performance in training and competition	Manifestation of aquaticity as a synergistic outcome of all domains
Training and diagnostic elements	Situational tasks (e.g., SAST, DAST, repeated dives, maximal performances)	Indirect manifest indicators of the level of aquaticity

**Table 2:** Hierarchical organization of aquaticity domains in breath-hold diving. Physiological regulation and neurophysiological control define the tolerance limits and stability of the system. Energetic aquaticity represents a functional intermediary responsible for the accumulation, distribution, and utilization of aerobic–anaerobic energy resources under breath-hold conditions. Biomechanical efficiency and perceptual–mental stability determine the execution quality and efficiency of movement, jointly shaping performance in aquatic sports.

Hierarchical Organization of Aquaticity Domains	
*Physiological Regulation* (tolerance limits, cardiorespiratory control)	*Neurophysiological Control* (sympathovagal balance, motor coordination)
*ENERGETIC AQUATICITY* (accumulation, distribution and utilization of aerobic–anaerobic energy resources)	
*Biomechanical Efficiency* (hydrodynamics, minimal energy cost)	*Perceptual–Mental Stability* (breathing urge control, focus, calmness)
Performance in Aquatic Sports	

## Data Availability

The original contributions presented in this study are included in the article. Further inquiries can be directed to the corresponding author.
